# Drug-induced esophageal injuries with an atypical presentation mimicking acute coronary syndrome

**DOI:** 10.1186/s12876-021-02063-2

**Published:** 2021-12-20

**Authors:** Guda Merdassa Roro, Geir Folvik, Liu Louis, Abate Bane

**Affiliations:** 1 Division of Gastroenterology, Department of Internal Medicine, College of Health Sciences, Addis Ababa University, Addis Ababa, Ethiopia; 2 Division of Gastroenterology, Department of Medicine, Haukeland University Hospital, Bergen, Norway; 3 Gastroenterology, Sinai Health System and University Health Network, University of Toronto, Toronto, Canada

**Keywords:** Drug-induced, Doxycycline, Pill-induced, Esophagitis, Esophageal injury, Acute coronary syndrome, Mimicking

## Abstract

**Background:**

Pill-induced esophageal injury may cause severe complications if not diagnosed in a timely fashion. The condition is under-recognized and under-reported, and some patients present with atypical clinical or endoscopic features mimicking other common conditions. If the diagnosis is missed the patient will continue to take the offending drug, potentially worsening the illness. We present a case in which acute coronary syndrome was the initial working diagnosis leading to a delay in diagnosis of doxycycline-induced esophageal injury. The patient developed multiple esophageal ulcers and hemorrhage.

**Case presentation:**

A 50-year-old male driver with a history of hypertension and dyslipidemia was brought to the emergency department with complaints of severe retrosternal chest pain, vomiting, diaphoresis and syncope. On initial evaluation, acute coronary syndrome was considered due to the clinical presentation and history of cardiovascular risk factors. Electrocardiogram and serum troponins were normal. On the second day of his admission, the patient developed odynophagia and bloody vomitus. Esophagogastroduodenoscopy revealed extensive esophageal ulcerations with hemorrhage. The patient was taking Doxycycline capsules for an acute febrile illness. Doxycycline is the oral medication most commonly reported to cause esophageal injury. Doxycycline was discontinued, and the patient was treated with intravenous omeprazole and oral antacid suspension. The patient improved, was discharged after 6 days of hospitalization, and reported resolution of all symptoms at an outpatient follow-up visit 3 weeks later.

**Conclusion:**

Medication-induced esophageal injury can present with atypical symptoms mimicking acute coronary syndrome. This condition should be included in the initial differential diagnosis of patients presenting with acute chest pain, especially those taking oral medications known to cause esophageal injury.

## Background

Pill-induced esophagitis was first reported by Pemberton et al. in 1970 [1]. Since then, more than one hundred different oral medications have been reported to cause pill-induced esophageal injury [2]. However, as of 1999 only about 1,000 cases of medication-induced esophageal injury had been reported, suggesting that this important condition is under-recognized and under-reported [2–6]. Retrosternal chest pain is the most common symptom, but is not specific for esophageal disease, whereas odynophagia and dysphagia occur in only 20% to 40% of patients with this condition [4, 7]. Hence, other common causes of chest pain may be considered. This condition can also mimic other diseases including esophageal cancer [8]. Patients in whom the diagnosis of pill-induced esophageal injury is not considered early will typically continue taking the offending medication [4], and may develop life-threatening complications due to continued exposure to the drug [1, 3, 4]. Therefore, awareness of this condition must be improved to facilitate its early diagnosis and treatment [4]. This case report describes a patient with an initial clinical presentation that mimicked an acute coronary syndrome. After 2 days of hospitalization gastrointestinal hemorrhage developed, and the correct diagnosis of doxycycline-induced esophageal injury was made.

## Case presentation

A 50 years old male long-distance truck driver presented to our Hospital in Addis Ababa, Ethiopia with severe constant retrosternal chest pain, diaphoresis and vomiting of ingested matter for the previous two days. The patient had a history of hypertension and elevated blood cholesterol levels. He was brought to the emergency department after he experienced an acute loss of consciousness of short duration. He reported that, after an episode of severe chest pain, he was not aware of his surroundings and lost control over his truck for a few seconds. The truck went off the road but fortunately no one was injured. The patient had no previous history of heart disease, and no cough or pleuritic pain. There was no history of alcohol or cigarette use.

Physical examination was normal except for hypertension (blood pressure 160/100 mm Hg) and low-grade fever with axillary temperature of 37.5 °C. He was admitted to hospital. Initially he was investigated for an acute coronary syndrome. Echocardiogram findings and serum troponin levels were normal. On the second days of his admission, he experienced one episode of bloody vomiting. On further questioning, his physicians learned that the patient had pain on swallowing. They also discovered that he was taking ceftriaxone injections and Doxycycline 100 mg capsules twice per day for four days. The medications were prescribed in another health facility for a febrile illness. The patient had no prior history of esophageal disease.

**Fig. 1:**
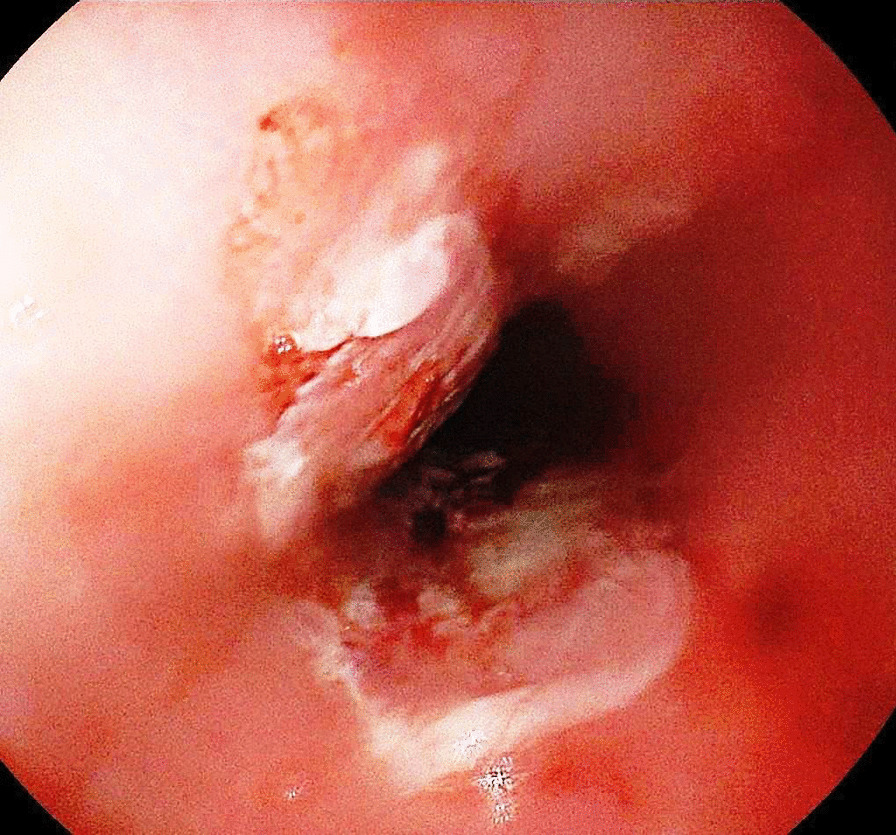
Typical “kissing ulcer” in upper esophagus

**Fig. 2:**
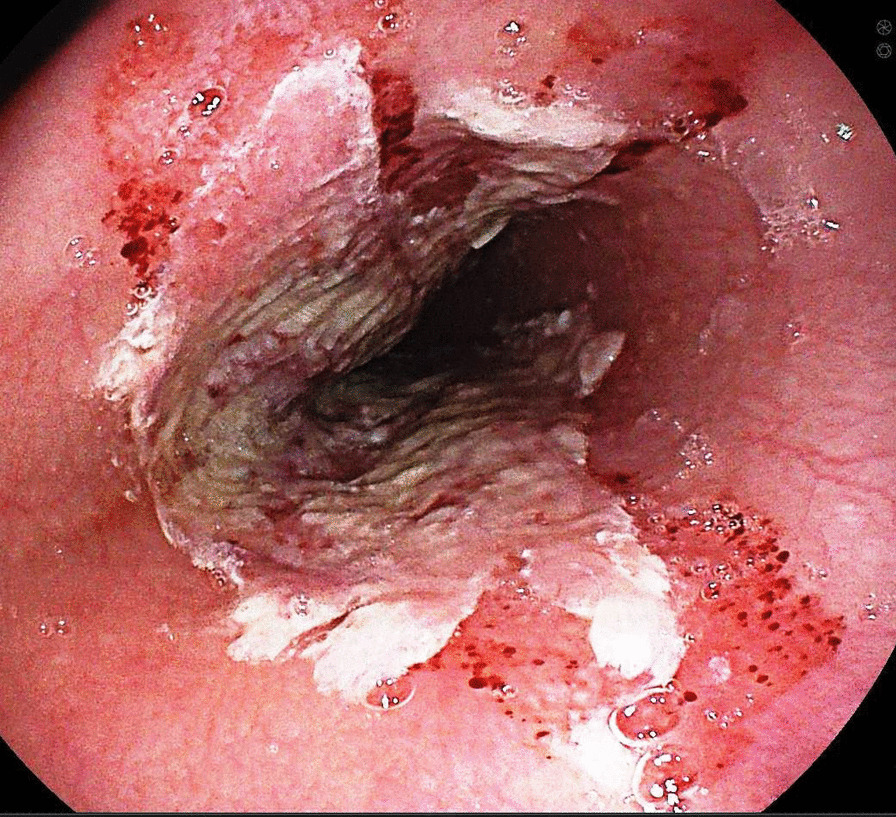
Pill-esophagitis in mid-Esophagus with remnant of the medication

A gastroenterologist was consulted and esophagogastroduodenoscopy (EGD) was performed. There were multiple mucosal ulcerations in the proximal and middle esophagus (Figs. 1 and 2) as well as at the lower esophageal sphincter. In addition, hyperemia and erosions were seen in both stomach and duodenum. Mild bleeding was noted.

Laboratory tests were normal except for a mild transient elevation of liver transaminases and a triglyceride level of 243 mg per deciliter. Serum albumin and bilirubin were normal. Hepatitis B surface antigen and Weil flex test were positive. Ultrasound of the abdomen showed increased echogenicity of the liver consistent with liver steatosis and fatty liver. There were no features of cirrhosis or portal hypertension. Chest X-ray, Complete blood count, blood film, and fasting blood sugar were all normal. H.Pylori stool antigen test, Hepatitis C. Virus and HIV antibody tests were negative. Diagnosis of Doxycycline-induced esophageal ulcerations was made, and doxycycline was discontinued. The patient was treated with ceftriaxone one gram intravenous twice daily to complete the course of treatment for the acute febrile illness and omeprazole 40 mg intravenous twice daily. He was also given antacid suspension orally. Parenteral analgesics were added as required. The pain and fever subsided gradually and the patient was discharged improved after 6 days, on omeprazole 20 mg orally twice daily for four weeks. He was advised to swallow pills in upright position and with water to prevent recurrence of similar problems. After 3 weeks, the patient returned for follow-up. He was asymptomatic. Liver transaminases were normal. Hepatitis B. Viral DNA level was 99 international unit per milliliter. Hepatitis e antigen was negative. Antiviral treatment was not indicated and the patient was linked to care for his hypertension and chronic hepatitis B infection.

## Discussion and conclusions

The most common oral drugs responsible for medication-induced esophagitis are antibiotics (36–60%), and doxycycline and tetracycline are the most frequent culprits [3, 4, 7]. Other common causes include non-steroidal anti-inflammatory drugs (35%), alendronate bisphosphonate, potassium chloride and antihypertensive drugs [2–4].

Most patient experience pill-induced injuries after ingesting the medication with little or no water shortly before they go to bed [2, 3]. Esophageal injury can be prevented by swallowing pills with an adequate amount of water (> 120 ml) while in an upright position, and staying in an upright position (sitting or standing) for 15 minutes after swallowing the medication [2, 3]. Other risk factors include old age, pre-existing esophageal disorders, enlarged left atrium and medication details (capsule formulation, bigger size, acidic and alkaline content) [2, 4, 9]. Our patient was taking a capsule formulation of doxycycline, the most commonly incriminated drug for causing drug-induced esophageal ulcers.

The middle segment of esophagus is the most common area injured (80%) as it is compressed by the aortic arch or an enlarged right atrium [2, 4]. Our patient had multiple ulcerations in different parts of the esophagus (upper, middle and at the level of the lower esophageal sphincter).

Diagnosis is mainly based on typical clinical manifestations of retrosternal chest pain (62–70%), odynophagia (40–79%), dysphagia (30–48%) and vomiting [2, 4, 5, 7]. Upper gastrointestinal endoscopy is considered the gold standard for diagnosis and is abnormal in 99% of affected patients [2, 7]. Biopsies are generally nonspecific and not helpful other than to exclude malignancy [2, 4, 9].

In our case, initially attention was given to investigations for acute coronary syndrome due to the patien’s risk profile and chief complaint of chest pain. The patient continued to take doxycycline until he developed hematemesis and was found to have odynophagia. These symptoms were the first clue to esophageal involvement and led to the decision to perform esophagogastroduodenoscopy.

In cases of delayed diagnosis, complications of pill esophagitis include esophageal bleeding, ulceration, penetration, perforation, stricture or even death [2–4]. Despite symptoms, our patient continued to take the drug for two days after hospitalization, and developed extensive ulcerations and bleeding.

Pill-induced esophagitis is managed by prompt discontinuation of the offending drug and administration of a PPI. Topical anesthetics, sucralfate and/or analgesics may also be part of the treatment [4]. In most cases, healing is complete with no or minimal scarring [4].

## Conclusion

Medication-induced esophageal injury is an important but often under-diagnosed condition. This is partly due to lack of awareness and partly because the clinical presentation can mimic other disorders like acute coronary syndrome. If not diagnosed early, it can potentially lead to serious complications and even death. The aim of this case report is to increase awareness among doctors and pharmacists on such atypical presentations.

## Data Availability

Not applicable. No datasets generated as this is a case report. The endoscopy pictures are available on request.

## Abbreviations

H.pylori: Helicobacter pylori
HIV: Human immunodeficiency virus
Mg: Milligram
MmHg: Millimeters of mercury
PPI: Proton pump inhibitor
